# Artisanal gold mine spoil types within a common geological area and their variations in contaminant loads and human health risks

**DOI:** 10.1007/s10661-023-10932-4

**Published:** 2023-01-20

**Authors:** Martin Kofi Mensah, Carsten Drebenstedt, Nils Hoth, Ibukun Momoriola Ola, Precious Uchenna Okoroafor, Edward Debrah Wiafe

**Affiliations:** 1 Institute of Surface Mining and Special Civil Engineering, Freiberg Technical University of Mining, Gustav-Zeuner Street 1A, Freiberg, 09599 Germany; 2 Institute of Biosciences/Interdisciplinary Environmental Research Centre, Freiberg Technical University of Mining, Leipziger Street 29, Freiberg, 09599 Germany; 3 School of Natural and Environmental Sciences, University of Environment and Sustainable Development, PMB Somanya, Ghana

**Keywords:** Artisanal mining, Potentially toxic elements, Mine spoil, Contamination, Human health risks, Sustainability

## Abstract

This study answered the question of whether mine spoils occurring in a common geological location had similarities in their contaminant load and associated health risks. Using inductively coupled plasma mass spectrometry, the total contents of Cd, Pb, As, Hg, Zn, Fe, and Al were determined for 110 digested soil samples obtained from underground rock ore (URS), oxide ore (OXS), and alluvial ore (AVS) mine spoils. Independent sample Kruskal–Wallis test and pairwise comparisons of sources were used to ascertain the variation in elemental load between the mine spoil investigated. The results showed that mine spoil contaminations and their ecological and health risk significantly varied (*p* < 0.01) from each other and fell in the order OXS > URS > AVS > forest soils because of their geochemistry. Determined enrichment and geo-accumulation indices revealed that OXS and URS sites were severely–extremely polluted with Cd, Hg, and As, while AVS mine spoils were only moderately contaminated by Cd and As contents. Children had the highest tendency for developing noncarcinogenic health defects largely due to toxic contents of As, Cd, and Hg in soil materials near them than adult men and women would after obtaining a hazard index of 73.5 and 67.7 (unitless) at both OXS and URS sites. Mine spoils especially where hard rocks and oxide ores were processed are not fit for agricultural use or human habitation. The restriction of human access and sustainable remediation approaches are required to avert health defects. Even so, area-specific potentially toxic elements must be targeted during soil cleaning due to the significant variations in contaminant load between mined sites.

**Supplementary Information:**

The online version contains supplementary material available at 10.1007/s10661-023-10932-4.

## Introduction

Potentially toxic elements (PTEs) in the ecosystem have gained increased attention worldwide due to their presence in the food chain and associated health effects (Li et al., 2018; Tóth et al., 2016a, b). Whereas their occurrence in the environment may be natural, anthropogenic pathways including pharmaceutical products, untreated wastewater discharges, agrochemicals, mining, and ore processing have significantly aggravated PTEs loads in the ecosystem (Deveci, 2013; Li et al., 2018; Soltani et al., 2017). Mine spoils are considered hotspots for PTEs such as Al, As, Pb, Ti, Cd, Fe, Mn, Ni, and V in the environments due to their binding association with ore bodies (Mensah et al., 2020a, b; Ramírez et al., 2020). Thus, their persistence makes them point sources for compromising the quality, safety, and integrity of surrounding environmental media such as plants, air, soil, and surface and groundwater resources (Affum et al., 2016; Hilson, 2002). However, since most PTEs are generally nondegradable, they become more toxic beyond their tolerable threshold levels and readily get absorbed in the tissues of living organisms causing human health risks (Zhang et al., 2010).

Many areas in Southern and Central Saxony, North Rhine-Westphalia, in Germany have predominantly high levels of PTEs in their soils due to mining and/or industrial use history, as some are not even safe for making children’s playgrounds, agriculture, or human habitation (Midula et al., 2017; Rinklebe et al., 2019; Tóth et al., 2016a, b). In Iran, varying contents of Cr, Cd, and Hg which were higher than their tolerable threshold levels were found near a silver mine surrounding (Soltani et al., 2017). Whiles in China alone, nearly 2 million hectares of contaminated lands at a rate of 46,600 ha/year were linked to the country’s massive industrialization drive (Xiao et al., 2017). The sequel is their severe contamination of terrestrial and aquatic habitats, and their products, a situation that causes dire food chain toxicities, and health defects and limits global efforts toward environmental sustainability (Soltani et al., 2017).

Elements such as Fe, Al, and As have established binding associations with gold ores and soil parent materials (Bundschuh et al., 2021). Their release and transfer either through natural (eruptions, wind, water) or anthropogenic (mining, agriculture, construction) increase the human health risk of PTE contamination through breathing, ingestion, dermal contacts, and/or consumption of contaminated food commodities (Bansah et al., 2016, 2018; Shaheen et al., 2020). Similarly, in Ghana, these risk factors may be higher due to the closeness of contaminated mine spoils to human installations such as homes, playgrounds, water bodies, and farms (Armah & Gyeabour, 2013; Bansah et al., 2018; Mensah et al., 2020a, b).

However, to date, the artisanal and small-scale mining (ASM) sector in Ghana rarely implements responsible raw materials extraction measures in their operations. This has led to widespread environmental degradation. The sequel is the generalized reports in the literature that mine spoils are toxic since they contain high contents of PTE that get released during the ASM mining processes. But the questions about whether all ASM spoils indeed have contaminant loads that are statistically equal or different remain unclarified by any scientific study. These knowledge shortfalls have created perceived complexities that limit key post-mining land use management decisions, especially in the areas of agriculture and habitation. It could, however, be assumed that since mine spoils are created from different ore forms, they may have distinct geochemical characteristics leading to differences in their contaminant load and associated health risk levels. Therefore, this study aimed at ascertaining the possible variation in the contents of potentially toxic elements (PTEs) that may exist in different mine spoils within common geolocation. The ecological and human health risks per mine spoil types were also estimated.

## Materials and methods

### Geology of study location

**Fig. 1:**
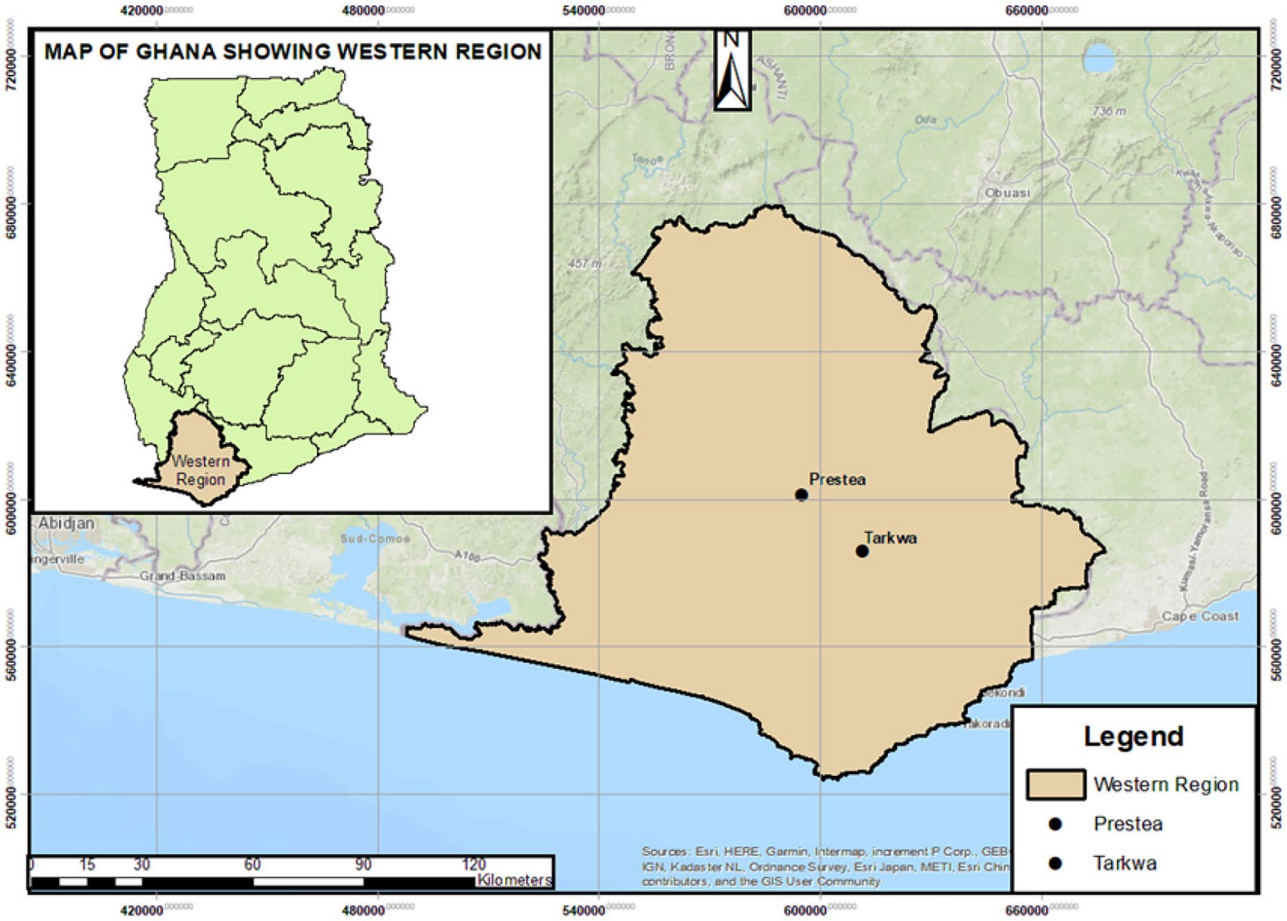
Map of Ghana indicating the study area

This investigation was undertaken between January 2020 and July 2022 within the Tarkwa-Prestea areas (05° 18′ 01″ N, 01° 58′ 01″ W and 05° 26′ 00″ N, 02° 08′ 00″ W in Southern Ghana) (Fig. 1). The geology of the area falls within the Paleoproterozoic Tarkwaian and Birimian group formations which encompass polymictic as well as quartz pebble conglomerates, sandstones and minor argillite, siltstones, and tuffs (Foli & Nude, 2012). Ferric acrisols also constitute the soil classification (Affum et al., 2016). Significant rivers in the area are the Ankobra and Bonsa rivers. The area which falls in the wet equatorial climate zone receives an average annual rainfall and temperature of about 1900 mm and 27 °C, respectively. According to Hayford et al. (2009), the soils are made up of quartz veins and sulfide ores formed after the weathering and oxidization of auriferous Birimian sedimentary rocks. The study area is noted for ASM prominence in the region and holds the highest number of large-scale mining operations in Ghana due to the richness of the gold ores (Owusu-Nimo et al., 2018).

### Sample management

#### Sampling and sample preparation

Random visitation to accessible but abandoned ASM sites was made for initial assessments. Based on the ore material mined, and resultant mine spoil properties, three groups of mine spoils were further investigated. For control purposes, independent composite soil samples from 20 different forest points nearby (> 1 km) of the mine sites were taken. During sampling, each large site was first divided into three parts; then random soil sampling at 0– 20 cm depth in a zigzag format was done using a soil auger to obtain a composite sample for each. Afterwards, each bulk sample was homogenized and subdivided to weigh about 3 kg before being taken to the laboratory. At the laboratory, they were sieved with a 2-mm mesh, homogenized, and subdivided to obtain a final representative fraction weighing about 500 g for air-drying, storage, and chemical analysis. Thus, triplicate samples from 30 different mined sites which were about > 500 m apart were investigated leading to a total sample size of 110 soils. The texture of the soils was determined directly on the field by wetting and feeling in the hand by a geoscientist; thereafter, sites with similar properties were grouped likewise for further assessment.

#### Laboratory analysis

Similar approaches used by Okoroafor et al. (2022) were employed to ascertain the target elemental concentrations in soils. Since soil samples were already homogenized and sieved, they were pulverized to help break any potential lumps that might have formed until they were about < 1 mm in size. Out of which, the 0.5-g analytic subsample was obtained. That is, 0.5 g of pulverized sample, together with 2 g each of Na_2_CO_3_ and K_2_CO_3_, was mixed, homogenized, and heated in a muffle furnace for 30 min at 900 degrees. Before cooling, liquefication with a 50-mL solution of 2-mol/L nitric acid and 0.5-mol/L citric acid was done; thereafter, ICP-MS (X series 2, Thermo Scientific) was used to measure the resultant solutions for their target elements. For quality control measurement, all samples were carefully measured in triplicates including triplicate portions of rhodium and rhenium solution at 10 ug/L. Soil pH and EC were determined for each sample after homogenized soil-to-deionized water at a ratio of 1:2.5 was made and measured with a well-calibrated PHS-3E pH meter and Eutech EcoTestr EC, respectively.

### Assessment of soil contamination and health risk indices

#### Soil contamination indices

Different indicators (Eqs. 1–3) were used to understand the varying extent of soil contamination due to the presence of the observed PTEs in this study. Using Eq. 1, we estimated the contamination factor (CF) to understand how contaminated the sites (Cs) were in comparison to their undisturbed state or worldwide averages for uncontaminated soils (Cref).

1 $$CF=\mathrm{Cs}/Cref (\mathrm{mg}/\mathrm{kg})$$

The reference background values for the various PTEs in mg/kg used were as follows: As = 6.8; Al = 20,000; Cd = 0.2; Hg = 0.01, Fe = 20,000; Pb = 27; and Zn = 70. Table 1 below grades the degree of mine spoil contamination based on the calculated values, while Eq. 2 was used to further understand whether the observed contaminations in the studied sites were due to human activities or naturally occurring (e.g., Antoniadis et al., 2017; Rinklebe et al., 2019).

**Table 1:** Interpretations of contamination and enrichment factors values for soils

Indices/range	Low/no contamination	Minor pollution	Moderately contamination	Considerable contamination	Very high contamination	Extremely polluted
CF range	< 1	–	1–3	3–6	> 6	–
EF range	< 1	1.5–3	3–5	–	5–10	> 10

2 $$EF=(Cs/Als)/(Cref/Alref)$$

where Als represented the total value of Al in this study and Alref, the worldwide value of Al in uncontaminated soils. The literary elements such as Fe and Al are generally geogenic so their values were therefore used as normalizer elements in estimating the spoil fortification levels in this estimation (Kobina et al., 2021; Li et al., 2015; Shaheen et al., 2020). Different interpretations (ranges) of EF values are presented in Table 1 as adopted from previous research (Antoniadis et al., 2017; Rinklebe et al., 2019). The estimation of the geo-accumulation index (*I*_*geo*_) using Eq. 3, helped in making a quantified assessment of the severity of the pollution matrix for a broader overview of the investigated mine spoils.

3 $$Igeo=\mathrm{log}2[CTE/1.5Cref]$$

The baseline concentration (Cref) was based on worldwide reference values while the 1.5 factor assisted to compliment any potential interference of background values due to human activities (Bempah & Ewusi, 2016; Mantey et al., 2020). Seven reference classes (Table 2) further helped to describe contaminant loads at the various sites.

**Table 2:** Grading scale for geo-accumulation index (*I*_*geo*_)

Index class	Unpolluted	Unpolluted to moderate pollution	Moderately polluted	Moderately to strong pollution	Strongly polluted	Strongly to extreme pollution	Extremely polluted
Class	Class 0	Class 1	Class 2	Class 3	Class 4	Class 5	Class 6
Range	*I*_*geo*_ ≤ 0	0 > *I*_*geo*_ ≤ 1	1 > *I*_*geo*_ ≤ 2	2 > *I*_*geo*_ ≤ 3	3 > *I*_*geo*_ ≤ 4	4 > *I*_*geo*_ ≤ 5	*I*_*geo*_ > 5

#### Human health risk indices

﻿During site visits, it was evident that there existed possibilities for humans to come in contact with target PTE due to their current unreclaimed, unrestricted access, and nearness to human installations and peoples’ ignorance about potential toxicities risks (Armah & Gyeabour, 2013; Bansah et al., 2018). Therefore, potential health risks to noncarcinogenic diseases were independently estimated for children, women, and men. This was aided by first calculating the average potential daily consumption or average daily dose (ADD) in mg/kg of body weight/day using Eq. 4 (eg., Bortey-Sam et al., 2015a, b; Kobina et al., 2021; Mensah et al., 2020a, b; Rinklebe et al., 2019; Shaheen et al., 2020).

4 $$ADD=\mathrm{Cs }((IR\times EF\times ED\times 10-6)/(BW \times AT))$$

Soil ingestion rate (*IR*) per day used was 100 and 200 mg/day for adults (matured females and men) and children, respectively. Exposure frequency (*EF*) was 250 and 350 days/year for adults and children. For exposure duration (*ED*), 25 and 6 years were assumed for both adults and children, respectively. Considering the obvious variation in body mass (BW) for human groups (all things being equal), 68-, 58-, and 15-kg weights were estimated for men, women, and children, respectively. For the average exposure period (AT) used, we multiplied the exposure length by the average number of days in a year (365). Thus ED* 365 culminates in 9125 and 2190 days for adults (men and women) and children, respectively, in their lifetime. The 10^–6^ factor helped with unit conversion only (Rinklebe et al., 2019). The calculated ADD values further assisted in estimating the hazard quotient (HQ) using Eq. 5 to understand the ratio of potential exposure to studied PTEs and the state at which no adverse health effects may occur.

5 $$HQ=ADD/RfD (\mathrm{mg}/\mathrm{kg})$$

The oral reference dose (*RfD*) for investigated PTEs used was As = 0.0003; Cd = 0.001; Fe = 0.7; Pb = 0.0035; Hg = 0.001, Al = 0.7; and Zn = 0.3, respectively. Lastly, all estimated *HQs* (Eq. 6) for all investigated PTEs per site were summed up to obtain the hazard index (HI) for adverse non-cancer health effects.

6 $$HI=HQ1+HQ2\dots \dots .HQn$$

The above health risk indices and reference dose used have previously been used extensively in the literature (e.g., Bortey-Sam et al., 2015a, b; Kobina et al., 2021; Mensah et al., 2020a, b; Rinklebe et al., 2019; Shaheen et al., 2020).

#### Statistical analyses

Using SPSS version 28 software, a nonparametric test for independent sample sources was made with the help of the independent sample Kruskal–Wallis test. This assisted to establish a general overview of how the distribution of target elements differed from each other. Additionally, pairwise comparisons of sources were done to ascertain potential site-by-site variation in potentially toxic element load at a 95% confidence level. Using Origin Pro 2022b software, box plots were created which showed contaminant mean and outliers. The error bars represented the standard deviation of samples.

## Results

### Artisanal gold ore processing methods

Generally, similar ore processing techniques are employed in Ghana’s ASM sector except for rock ore forms which required additional steps such as breaking and transportation before milling and washing. The oxide and alluvial ore forms were milled in situ with the help of a mining machine called Chan fa. The gold debris is then trapped with board and collectors for onward recovery using mercury to form a gold amalgam. But to obtain only the raw gold, heating is applied to release the Hg through vaporization. The effluent from all these processes is not trapped for treatment or recycling and, thus, freely moved into the environment. Mining and processing of gold were observed occurring at places such as within forest reserves, farms, within and near river bodies, and residential areas.

### Potentially toxic elements in mine spoil

**Fig. 2:**
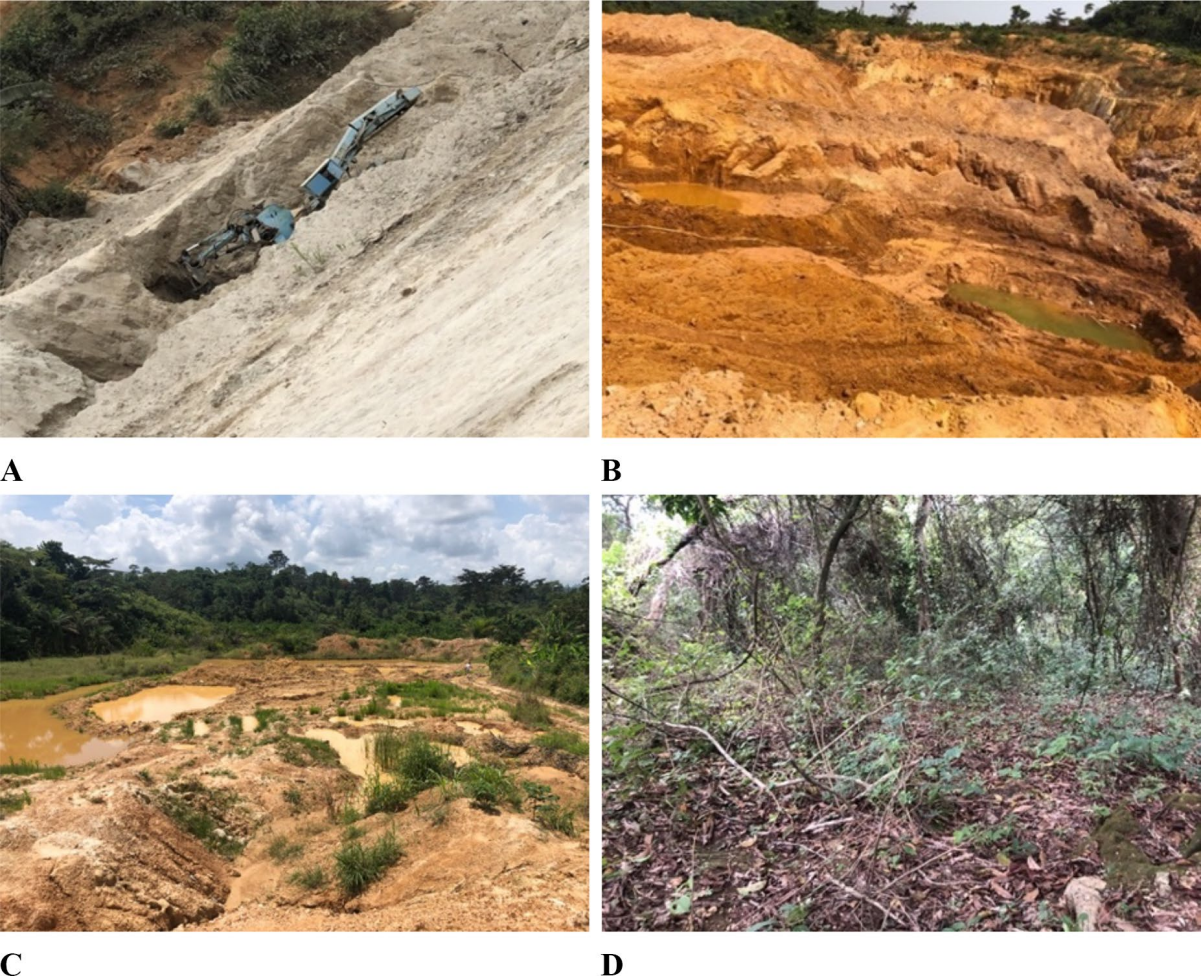
Physical characteristics of some sampling sites investigated. **A** Mine spoils from underground rock ores (URS), **B** mine spoils from an oxide gold ore deposit (OXS), **C** mine spoils from an alluvial gold ore deposit (AVS), and **D** forest area for control sampling (FS)

Despite the differences in the scale and ore materials mined, a mosaic landscape of inundated pond waters and patchy grasses were common features found in most mining sites. However, their physical properties were very distinct and predominantly sandy, silty-clay, and clayey-gravel as they reflected the type of ore materials mined and processed. That is, the three types of mine spoil identified and investigated included (i) the underground rock mine spoil (URS), which was characterized by pure sand generated after the mining and processing of gold present in sulfuric rocks beneath the earth’s crust, (ii) the oxide mine spoil (OXS), which was characterized by silty-clay materials created after the processing of free metallic gold in oxide earth materials, and (iii) the alluvial mine spoil (AVS), which was characterized by clayey-gravel mine spoils created after processing placer ore deposits for free metallic gold, respectively (Fig. 2). The sampled forest soils which served as the control points were however clay loam in texture. Most of the studied artisanal and small-scale mining sites (ASM) were observed to have hugely occurred within forests, farms, and near human settlements while the degraded lands were found to be barrierless, had unsafe landscapes, and were without signs of reclamation. Winning of sand from URS sites (Fig. 2A) and gravels from AVS sites (Fig. 2C) by nearby inhabitants for building construction was observed during site visits.

**Fig. 3:**
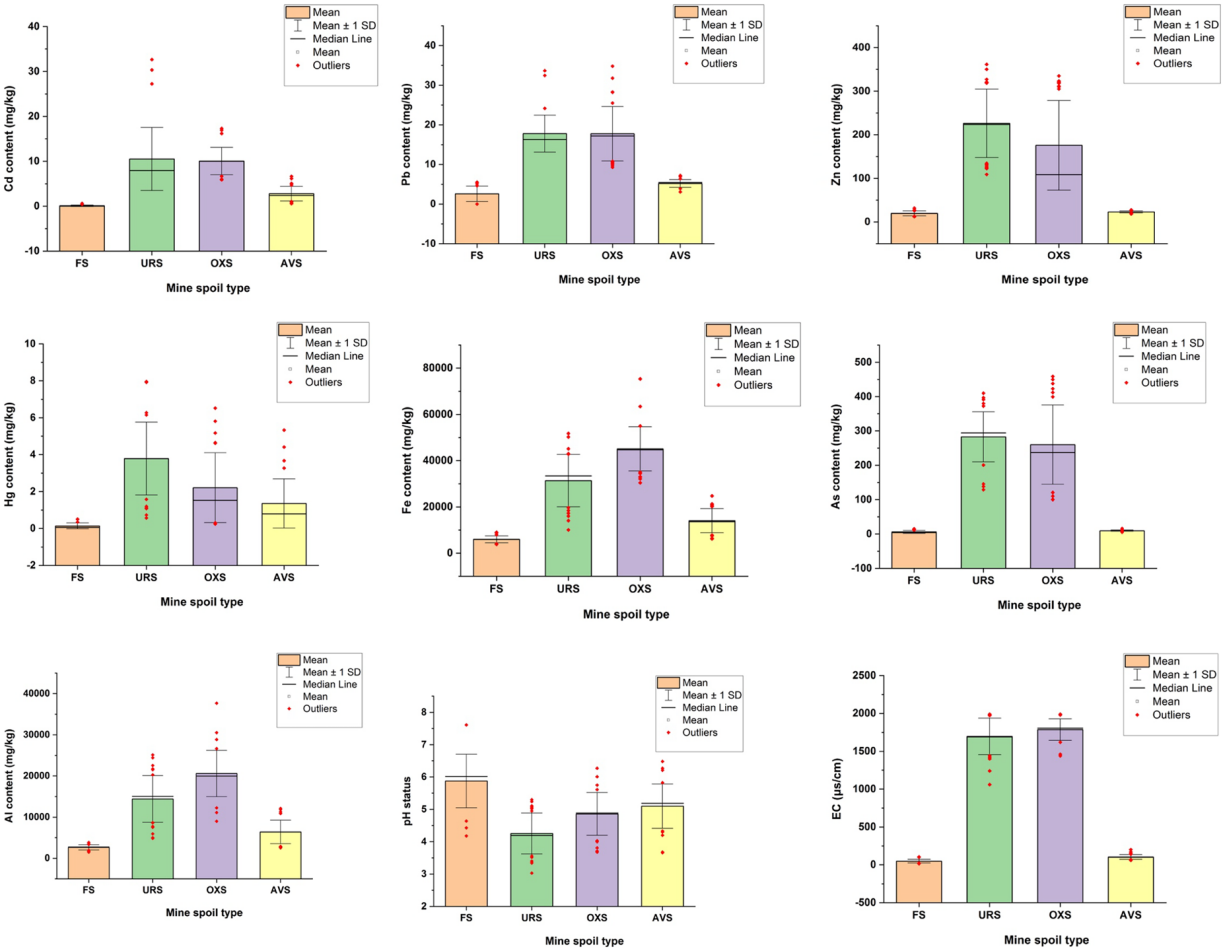
Box plots showing the mean total contents of potentially toxic elements in the investigated sites. Error bars indicate the standard deviation of samples. (FS, forest soil; URS, underground rock mine spoil; OXS, oxide mine spoil; AVS, alluvial mine spoil)

The observed mean ± standard deviation of background chemical properties for all mine spoil types and forest sites are presented in Fig. 3 below. All soil samples from both mining and forest sites were acidic whereas the average EC values varied largely in the range of 48.5–1786.0 μS/cm. Mine sites were more consolidated during sampling and had an average bulk density range of 1.36–1.83 g/cm^3^ while the forest soils had an average bulk density of 1.17 g/cm^3^. The obtained total Pb contents in all samples, then the total average contents of Cd, Hg, Fe, and Al in forest soils only, were within the tolerable threshold limits for agricultural soils (Kabata-Pendias, 2011). Based on the results from the independent sample Kruskal–Wallis test, the distribution of target PTEs across all sources was found to vary significantly (*p* < 0.01) from each other with the URS sites containing the highest total average contents of Cd, Pb, As, Zn, and Hg. Further, pairwise comparisons of the mine spoil revealed that the distribution of Cd, Zn, Pb, and As among alluvial mine spoil (AVS) and forest soil (FS) and that of underground mine spoil and oxide mine spoils were not significantly different (*p* > 0.05) from each other. For Hg and pH, only AVS and OXS sources were comparable (*p* > 0.05) with each other.

This study further established that nearly 40% of the individual forest site had total contents of As beyond the 6.8-mg/kg world soil average (WSA) even though their total average content of 5.9 ± 4.3 mg/kg (mean ± SD) was lower but slightly above the set TLV of 5.0 mg/kg. However, for the mine spoils, they all recorded 100% site contaminations for As which was about 1.4, 38, and 41 folds higher than the 5.0-mg/kg tolerable threshold value in agricultural soils for AVS, URS, and OXS, respectively. Even that, only the UrS and OxS spoils had their average total contents becoming 4-fold mored than the set 65.0 mg/kg/As trigger action value in soils. Again, while the average Zn contents across all sites exceeded the 70 mg/kg projected for undisturbed soils based on worldwide averages, they fell within the set tolerable threshold of 300 mg/kg for agricultural soils (Kabata-Pendias, 2011; Kobina et al., 2021).

### Soil contamination indices

The relatively higher total contents of most PTEs investigated resulted in a sequel contamination factor (*CF*) value which was above unity for most sites. For instance, apart from Pb and Al, all the remaining PTEs in the URS and OXS spoils had their average total contents contributing to a *CF* > 1. In the case of AVS spoils, only its Cd and Hg contents resulted in moderate contaminations while forest soils remained uncontaminated with the studied PTEs except for Hg at *CF* = 1.4 mg/kg. Cumulatively, the URS sites recorded the highest enrichments values which were followed by the OXS sites due to their high contents of Cd, Hg, and As. The total average contents of Cd and Hg in AVS sites and Hg only in forest soils contributed to severe soil enrichments (Table 3), with the remaining PTEs either causing minor (1.5—3.0) or no contaminations (< 1.5). The estimated *I*_*geo*_ (Table 4) for the various PTEs revealed significant human-induced contaminations in all mining sites with the highest influences occurring at URS and OXS sites.

**Table 3:** Soil contamination factor (*CF*) for various sample sources

Spoil type	Potentially toxic elements (mg/kg)						
	Cd	As	Pb	Hg	Fe	Zn	Al
FS	0.5	0.9	0.1	1.4^c^	0.3	0.3	0.1
UrS	47.5^a^	41.6^a^	0.7	37.9^a^	1.6^c^	3.2^b^	0.3
OxS	50.2^a^	38.3^a^	0.7	22.1^a^	2.3^c^	2.5^c^	1.0^c^
AvS	13.9^a^	1.4^c^	0.2	13.5^a^	0.7	0.3	0.7

^a^Very high contamination

^b^Considerable contamination

^c^Moderate contamination

**Table 4:** Geo-accumulation estimation (*I*_*geo*_) for studied sample sources

Sample source	Potentially toxic elements (mg/kg)				
	Cd	As	Pb	Hg	Zn
FS	− 1.6	− 0.8	− 4.0^b^	− 0.1	− 2.4
UrS	5.0^a^	4.8^b^	− 1.2	4.7	1.1
OxS	5.1^a^	4.7^b^	− 1.2	3.9^c^	0.7
AvS	3.2^c^	− 0.1	− 3.0	3.2^c^	− 2.2

^a^Very high contamination

^b^Considerable contamination

^c^Moderate contamination

### Human health risk indices

Apart from Fe and Al total contents which were not considered for health risk assessment, the remaining PTEs (Cd, As, Pb, Hg, and Zn) held varying potentials to cause health defects at the mine spoil sites. Meanwhile varying health risks to matured females and males per 10,000 population were observed for all mine spoils due to the prevailing PTE levels. Children who may have regular access to URS and OXS mine spoils were also exposed to hazard quotients (HQs) of 66 and 64, respectively (*Table* S2–S4*: supplementary material*). This was influenced by their extremely severe As content further culminated in a very high hazard index (*HI*) of 78.5 and 74.9 (unitless) for children among all 7 PTEs investigated as shown in Table 5. However, AVS and forest sites had *HI* values < 6 for children. The forest soils were found to be the least contributing to HQs and *HI* in all human groups. Additionally, both matured females and males had *HI* < 7.5 (unitless) for all sites investigated as arsenic alone contributed more than 50% of this total *HI* value. Cumulatively, the *HI* hierarchy of URS > OXS > AVS > FS was obtained across all sites in this present study.

**Table 5:** Hazard index (*HI*) to human groups

Sample source	Human groups (unitless)		
	Child	Men	Women
FS	1.6	0.1	0.1
UrS	73.5	5.8	6.8
OxS	67.7	5.3	6.3
AvS	2.8	0.2	0.3

(*HI* values > 1 indicate a high potential for noncarcinogenic health disorders)

## Discussion

### Mine spoil contaminations

Categorizing mine spoils based on their physicochemical characteristics can provide essential knowledge for post-mining land use management decisions, especially when considerations for agriculture or human habitations are involved. Despite this study’s limitations of not subjecting the soil parent materials to thorough geochemical analyses other than the total contents of target PTEs in this study, the results revealed how ASM spoils within a common geological area could be distinctly grouped. The tolerable threshold levels (Hg = 0.5 mg/kg, As = 5.0 mg/kg, Zn = 200 mg/kg, Pb = 150 mg/kg, Cd = 1.0 mg/kg, Fe = 20,000, Al = 20,000) and trigger action values (1.5 mg/kg, 10 mg/kg, 200–1500 mg/kg, and 2 mg/kg for Hg, As, Zn, and Cd, respectively) used for further discussion in this study are based on the European soil guidelines and the WHO as used by previous authors (e.g., Kabata-Pendias, 2011; Tóth et al., 2016a, b).

According to Kobina et al. (2021) and Hayford et al. (2009), arsenopyrite-linked gold ores make up larger portions of the total gold crop in southern Ghana. Therefore, through the oxidations of As(III) to FeAsS (arsenopyrite), the discharge of sulfuric acids and iron oxyhydroxides occurs in the environment (Armah et al., 2014; Xiao et al., 2017). This proven process might have been the reason for the generally low pH and high Fe substrates in mine spoil and forest soils as gathered by this study (Foli & Nude, 2012; Kabata-Pendias, 2011). Additionally, at low pH, favorable conditions for oxidative reactions of geogenic As are noted to further release trace elements such as Cd, Pb, Pb, Cd, Cu, Co, and Zn into the environment (Dzigbodi-Adjlmah, 1993; Foli & Nude, 2012; Kobina et al., 2021; Simion et al., 2020). For instance, finding average Fe contents of 5974.1 mg/kg in forest soils was consistent with earlier research that high quantities of Fe in the form of FeAsS and FeAsO_4_ were fundamental components in most Ghanaian soils (Hayford et al., 2009; Kobina et al., 2021; Mensah et al., 2020a, b). This assertion was made after finding about 6,208–60,320 mg/kg and 2350–3232 mg/kg of Fe in abandoned mine spoils and in forest areas in our study region (Kobina et al., 2021). Thus, the above conditions might be the largest reason for the presence of varying contents of PTEs obtained in this study, and we assume that they may be significant pathways for further contaminating surface and groundwater systems through acid mine drainages.

Notwithstanding, the large differences in PTE load between mine types and forest soils showed the extent to which human-induced weathering which includes excavation and milling of earth materials as well as their respective geochemical characteristics of each site, might have influenced their abundance in areas where they occur (Guan et al., 2014). In this study, the total contents of Fe, Al, and then As, in both URS and OXS sites which have binding affiliations to gold as stated by Bundschuh et al. (2021) exceeded the 20,000 and 5 mg/kg maximum background contents in natural soils, respectively. Thus, the highest total arsenic contents of 282.8 mg/kg observed at URS sites exceeded the set worldwide averages in uncontaminated soils (2 mg/kg), threshold limit values in agriculture soils (5 mg/kg), and the trigger action values in the range 10–50 mg/kg (Kabata-Pendias, 2011). The implication of high contents of Fe and As in the environment for instance could be their possible movement and subsequent contaminations of both surface and groundwater resources. When this occurs, incidents of their related health disorders might be magnified among communities. Despite this, our observed As content was far lower than the 8,900— 30,700 mg/kg of As recorded near large-scale mine vicinities in the Bolivian Andes, Northwest Spain, and Ghana (Larios et al., 2012; Martínez-Sánchez et al., 2011; Mensah et al., 2020a, b).

Since according to Affum et al. (2016) cinnabar (HgS) naturally occurs in rock materials in non-generating in our study areas, it is implied that the observed Hg contaminations were mainly from anthropogenic sources. In the artisanal mining environment, the use, poor handling, storage, and recovery of industrial Hg used for gold processing are largely known to be the main pathways for Hg suppliers into the environment. These occur through processes such as Hg, e.g., vaporization and condensation, untreated waste flows, and leakages into the environment (Bempah & Ewusi, 2016; Mantey et al., 2020). These might be the reason for the obtained traces of Hg in forest soils even though their long distances (> 1 km) from gold processing centers and the buffering properties of the forest’s dense vegetation might have reduced their total contents (Mensah et al., 2020a, b). Mercury contaminations in Ghanaian near mining areas are widely speculated to be more grave than this study established and might be due to over emphasization of the problem or that the pollution loads were reduced through Hg volatilization, water dissolution, and leaching due to the age of the abandoned mine spoils. Despite these, the ascertained Hg contamination levels across all mine spoils studied are still enough to cause health disorders unless remediated.

For the observed total contents of Cd (2.8—10 mg/k) in the various mine spoils investigated, two possible sources might have contributed to contaminations as they exceeded the respective background values in uncontaminated (0.41 mg/kg) and tolerable limits value (1.0 mg/kg) in soils (Kabata-Pendias, 2011). These may include either residual contaminants from previously used farm inputs (fertilizers and pesticides) due to the pre-mining land use for farming or from mining-enhanced weathering and disposition from geogenic sources.

Whereas our reported Cd contents require cleanups owing to their potential to cause toxicities, they were, however, far lower than the 270, 468, 1500, and 1781 mg/kg Cd found near mining and processing areas in Poland, Great Britain, the USA, and Belgium, respectively (Kabata-Pendias, 2011). Unless reclaimed which was not the case in the various sites investigated, their persistence in the environment could contaminate food crops as widely known in the literature including those found in turnip leaves (0.5 mg/kg), spinach (6.4 mg/kg), lichens (22.0 mg/kg), and lettuce (45 mg/kg) in Germany, Zambia, Belgium, and Australia, respectively (Bortey-Sam et al., 2015a, b; Hecker, 2005; Kabata-Pendias, 2011; Soltani et al., 2017).

### Soil contamination indices

Highly mineralized and reactive rock and oxide materials were found to hold higher contents of investigated PTEs that caused extreme pollution in URS and OXS sites. Thus, such lands could potentially lead to high ecological risk through the food chain and groundwater contamination much more than the AVS and forest sites would. Contents of Cd in turnip leaves (0.5 mg/kg), spinach 6.4 mg/kg), lichens (22.0 mg/kg), and lettuce (45 mg/kg) were reported around mine sites in Germany, Zambia, Belgium, and Australia, respectively (Bortey-Sam et al., 2015a, b; Hecker, 2005; Kabata-Pendias, 2011; Soltani et al., 2017). With similar ecotoxicological risks projected in the nearest future owing to the presence of the observed PTE hazards, the remediation of mine spoils could assist in reducing potential food chain contaminations (Mensah et al., 2022).

### Human health risk indices

Owing to these, higher chances exist that children near UrS and OxS sites might develop noncarcinogenic health risks than adult men and women would in the same vicinities (Table 5). The sequel to high PTE contents in mine soils is increased human health risks. Owing to these, higher chances exist that children near URS and OXS sites might develop noncarcinogenic health risks than adult men and women would in the same vicinities. This might be largely caused by the high As toxicities (*HQ* = 74.5 and 68.0 for OXS and URS, respectively) due to its reactiveness. Additionally, since arsenic has been identified as a class 1 carcinogen, its extreme presence in the environment can increase human risks of cancer development (Bortey-Sam et al., 2015a, b; Mensah et al., 2020a, b). These may occur through dermal contact or ingestion or consuming contaminated food and water from contaminated areas (Kobina et al., 2021). Lower possibilities (HI < 1) were, however, not established for adult men or women living near localities where AVS or FS sites and such areas have lower possibilities to cause health defects except for children. This observation thus agrees with available knowledge that children stand higher chances of soil toxicities than adults due to their easiness of accidentally ingesting contaminants (Mensah et al., 2020a, b; Shi et al., 2014). However, the observed winning of contaminated mine materials for construction, the presence of arable farms near mining areas, and the proximity of human settlements to mine spoil present increased human contact with PTEs that could accelerate health risks to all human groups. In such instances, common human health implications of PTE contaminations have been comprehensively reported in the literature (e.g., Ali et al., 2013; Basu et al., 2015; Obiri et al., 2010; Quansah et al., 2015; Rajaee et al., 2015a, b) and may rampantly arise in host mining communities. Mensah et al. (2020b) reported how gold mine workers were exposed to mine contaminants through breathing at their workplace. Artisanal gold processing in the homes of communities in Northwestern Nigeria was also linked to the cause of child mortalities due to lead poisoning from their activities (Dooyema et al., 2012).

## Conclusion

This study revealed how artisanal gold mine spoils could be distinctly grouped into three (3) types to include underground rock mine spoils (URS), oxide mine spoils (OXS), and then alluvial mine spoils (AVS) as influenced by their ore and spoil physical properties. However, their level of pollution load and associated health risks are linked to the geochemistry of the material type mined in an area. The order of contamination and health risk established was OXS > URS > AVS > FS among sites with contents of As, Hg, and Cd contributing to severe–extreme soil contaminations. Additionally, the likelihood that more children might develop noncarcinogenic health diseases near mine spoils existed highly at the OXS and URS sites than at the AVS sites. Additionally, the occurrence of massive degradation of forests and farmlands through unsustainable mining threatens ecosystem sustainability and increases climate change effects and food insecurities.

Since humans had increased contact rates with these polluted mine materials due to their proximity and easy access to contaminated mine spoils, an urgent need for community education is required while As, Hg, and Cd-related diseases surveillance should be made. Also, remediation of these abandoned mine lands is encouraged to control contaminant transport and avert their ripple effects on human health. This study believes that areas, where URS and OXS mine spoils exist hold higher ecological and human health risks and that is unfit for agriculture, habilitation, or recreation unless sustainably remediated.

## Supplementary Information

Below is the link to the electronic supplementary material.

Supplementary file1 (ZIP 1654 KB)

## Data Availability

All data supporting the findings of this study in its current form are available within the paper and supplied supplementary materials.

## References

[CR1] Affum AO, Dede SO, Nyarko BJB, Acquaah SO, Kwaansa-Ansah EE, Darko G, Dickson A, Affum EA, Fianko JR (2016). Influence of small-scale gold mining and toxic element concentrations in Bonsa river, Ghana: A potential risk to water quality and public health. Environmental Earth Sciences.

[CR2] Ali H, Khan E, Sajad MA (2013). Phytoremediation of heavy metals—Concepts and applications. Chemosphere.

[CR3] Antoniadis V, Shaheen SM, Boersch J, Frohne T, Du Laing G, Rinklebe J (2017). Bioavailability and risk assessment of potentially toxic elements in garden edible vegetables and soils around a highly contaminated former mining area in Germany. Journal of Environmental Management.

[CR4] Armah FA, Gyeabour EK (2013). Health risks to children and adults residing in riverine environments where surficial sediments contain metals generated by active gold mining in Ghana. Toxicological Research.

[CR5] Armah FA, Quansah R, Luginaah I (2014). A systematic review of heavy metals of anthropogenic origin in environmental media and biota in the context of gold mining in Ghana. International Scholarly Research Notices.

[CR6] Bansah KJ, Dumakor-Dupey NK, Stemn E, Galecki G (2018). Mutualism, commensalism or parasitism? Perspectives on tailings trade between large-scale and artisanal and small-scale gold mining in Ghana. Resources Policy.

[CR7] Bansah KJ, Yalley AB, Dumakor-Dupey N (2016). The hazardous nature of small scale underground mining in Ghana. Journal of Sustainable Mining.

[CR8] Basu N, Clarke E, Green A, Calys-Tagoe B, Chan L, Dzodzomenyo M, Fobil J, Long RN, Neitzel RL, Obiri S, Odei E, Ovadje L, Quansah R, Rajaee M, Wilson ML (2015). Integrated assessment of artisanal and small-scale gold mining in Ghana-part 1: Human health review. International Journal of Environmental Research and Public Health.

[CR9] Bempah, C. K., & Ewusi, A. (2016). Heavy metals contamination and human health risk assessment around Obuasi gold mine in Ghana. *Environmental Monitoring and Assessment*, *188*(5). 10.1007/s10661-016-5241-3 10.1007/s10661-016-5241-3 27037696

[CR10] Bortey-Sam N, Nakayama SMM, Akoto O, Ikenaka Y, Fobil JN, Baidoo E, Mizukawa H, Ishizuka M (2015). Accumulation of heavy metals and metalloid in foodstuffs from agricultural soils around Tarkwa area in Ghana, and associated human health risks. International Journal of Environmental Research and Public Health.

[CR11] Bortey-Sam N, Nakayama SMM, Ikenaka Y, Akoto O, Baidoo E, Yohannes YB, Mizukawa H, Ishizuka M (2015). Human health risks from metals and metalloid via consumption of food animals near gold mines in Tarkwa, Ghana: Estimation of the daily intakes and target hazard quotients (THQs). Ecotoxicology and Environmental Safety.

[CR12] Bundschuh, J., Schneider, J., Alam, M. A., Niazi, N. K., Herath, I., Parvez, F., Tomaszewska, B., Guilherme, L. R. G., Maity, J. P., López, D. L., Cirelli, A. F., Pérez-Carrera, A., Morales-Simfors, N., Alarcón-Herrera, M. T., Baisch, P., Mohan, D., & Mukherjee, A. (2021). Seven potential sources of arsenic pollution in Latin America and their environmental and health impacts. *Science of the Total Environment*, *780*. 10.1016/j.scitotenv.2021.146274 10.1016/j.scitotenv.2021.146274 34030289

[CR13] Deveci, T. (2013). Assessment of trace element concentrations in soil and plants from cropland irrigated with wastewater. *Ecotoxicology and Environmental Safety*, *98*. 10.1016/j.ecoenv.2013.08.013 10.1016/j.ecoenv.2013.08.013 24021872

[CR14] Dooyema, C. A., Neri, A., Lo, Y., Durant, J., Dargan, P. I., & Swarthout, T. (2012). *Research | Children’s health outbreak of fatal childhood lead poisoning related to artisanal gold*. *120*(4), 601–607 10.1289/ehp.1103965 PMC3339453 22186192

[CR15] Dzigbodi-Adjlmah K (1993). Geology and geochemical patterns of the Birimian gold deposits. Journal of Geochemtcal Exploratton.

[CR16] Foli G, Nude PM (2012). Concentration levels of some inorganic contaminants in streams and sediments in areas of pyrometallurgical and hydrometallurgical activities at the obuasi gold mine. Ghana. Environmental Earth Sciences.

[CR17] Guan Y, Shao C, Ju M (2014). Heavy metal contamination assessment and partition for industrial and mining gathering areas. International Journal of Environmental Research and Public Health.

[CR18] Hayford EK, Amin A, Osae EK, Kutu J (2009). Impact of gold mining on soil and some staple foods collected from selected mining communities in and around tarkwa-prestea area. West African Journal of Applied Ecology.

[CR19] Hecker, R. (2005). Trace elements. In *Packaging Magazine* (Vol. 8, Issue 13).

[CR20] Hilson G (2002). The environmental impact of small-scale gold mining in Ghana: Identifying problems and possible solutions. Geographical Journal.

[CR21] Kabata-Pendias A. (2011). Trace elements in soils and plants. In *Taylor & Francis Group, Boca Raton London New York.* (4th ed.). Taylor & Francis Group, Boca Raton London New York. 10.1201/b10158

[CR22] Kobina A, Marschner B, Antoniadis V, Stemn E, Shaheen SM, Rinklebe J (2021). Science of the total environment human health risk via soil ingestion of potentially toxic elements and remediation potential of native plants near an abandoned mine spoil in Ghana. Science of the Total Environment.

[CR23] Larios R, Fernández-Martínez R, Rucandio I (2012). Comparison of three sequential extraction procedures for fractionation of arsenic from highly polluted mining sediments. Analytical and Bioanalytical Chemistry.

[CR24] Li L, Wu J, Lu J, Min X, Xu J, Yang L (2018). Distribution, pollution, bioaccumulation, and ecological risks of trace elements in soils of the northeastern Qinghai-Tibet Plateau. Ecotoxicology and Environmental Safety.

[CR25] Li P, Lin C, Cheng H, Duan X, Lei K (2015). Contamination and health risks of soil heavy metals around a lead/zinc smelter in southwestern China. Ecotoxicology and Environmental Safety.

[CR26] Mantey J, Nyarko KB, Owusu-Nimo F, Awua KA, Bempah CK, Amankwah RK, Akatu WE, Appiah-Effah E (2020). Mercury contamination of soil and water media from different illegal artisanal small-scale gold mining operations (galamsey). Heliyon.

[CR27] Martínez-Sánchez MJ, Martínez-López S, García-Lorenzo ML, Martínez-Martínez LB, Pérez-Sirvent C (2011). Evaluation of arsenic in soils and plant uptake using various chemical extraction methods in soils affected by old mining activities. Geoderma.

[CR28] Mensah, A. K., Marschner, B., Shaheen, S. M., Wang, J., Wang, S. L., & Rinklebe, J. (2020a). Arsenic contamination in abandoned and active gold mine spoils in Ghana: Geochemical fractionation, speciation, and assessment of the potential human health risk. *Environmental Pollution*, *261*. 10.1016/j.envpol.2020.114116 10.1016/j.envpol.2020.114116 32220748

[CR29] Mensah K, M., Drebenstedt, C., Okoroafor, P. U., & Wiafe, E. D. (2022). Jatropha curcas and Manihot esculenta are super plants for phytoremediation in multi-contaminated mine spoils. *International Multidisciplinary Symposium “UNIVERSITARIA SIMPRO 2022”: Quality and Innovation in Education, Research and Industry – the Success Triangle for a Sustainable Economic, Social and Environmental Development*, *373*, 1–8. 10.1051/matecconf/202237300080

[CR30] Mensah MK, Mensah-Darkwa K, Drebenstedt C, Annam BV, Armah EK (2020). Occupational respirable mine dust and diesel particulate matter hazard assessment in an underground gold mine in Ghana. Journal of Health and Pollution.

[CR31] Midula, P., Wiche, O., Wiese, P., & Andráš, P. (2017). *Concentration and bioavailability of toxic trace elements, germanium, and rare earth elements in contaminated areas of the Davidschacht dump-field in Freiberg (Saxony)*. *April*.

[CR32] Obiri S, Dodoo DK, Armah FA, Essumang DK, Cobbina SJ (2010). Evaluation of lead and mercury neurotoxic health risk by resident children in the Obuasi municipality. Ghana. Environmental Toxicology and Pharmacology.

[CR33] Okoroafor, P. U., Ogunkunle, C. O., Heilmeier, H., & Wiche, O. (2022). Phytoaccumulation potential of nine plant species for selected nutrients, rare earth elements (REEs), germanium (Ge), and potentially toxic elements (PTEs) in soil. *International Journal of Phytoremediation*, *0*(0), 1–11. 10.1080/15226514.2021.2025207 10.1080/15226514.2021.2025207 35014898

[CR34] Owusu-Nimo, F., Mantey, J., Nyarko, K. B., Appiah-Effah, E., & Aubynn, A. (2018). Spatial distribution patterns of illegal artisanal small scale gold mining (Galamsey) operations in Ghana: A focus on the Western Region. *Heliyon*, *4*(2). 10.1016/j.heliyon.2018.e00534 10.1016/j.heliyon.2018.e00534 PMC5835009 29511743

[CR35] Quansah R, Armah FA, Essumang DK, Luginaah I, Clarke E, Marfoh K, Cobbina SJ, Edward N-A, Namujju PB, Obiri S, Dzodzomenyo M (2015). Association of arsenic with adverse pregnancy outcomes/infant mortality. Enviromental Health Perspectives.

[CR36] Rajaee M, Obiri S, Green A, Long R, Cobbina SJ, Nartey V, Buck D, Antwi E, Basu N (2015). Integrated assessment of artisanal and small-scale gold mining in Ghana—part 2: Natural sciences review. International Journal of Environmental Research and Public Health.

[CR37] Rajaee M, Sánchez BN, Renne EP, Basu N (2015). An investigation of organic and inorganic mercury exposure and blood pressure in a small-scale gold mining community in Ghana. International Journal of Environmental Research and Public Health.

[CR38] Ramírez O, Sánchez de la Campa AM, Sánchez-Rodas D, de la Rosa JD (2020). Hazardous trace elements in thoracic fraction of airborne particulate matter: Assessment of temporal variations, sources, and health risks in a megacity. Science of the Total Environment.

[CR39] Rinklebe J, Antoniadis V, Shaheen SM, Rosche O, Altermann M (2019). Health risk assessment of potentially toxic elements in soils along the Central Elbe River, Germany. Environment International.

[CR40] Shaheen, S. M., Antoniadis, V., Kwon, E., Song, H., Wang, S. L., Hseu, Z. Y., & Rinklebe, J. (2020). Soil contamination by potentially toxic elements and the associated human health risk in geo- and anthropogenic contaminated soils: A case study from the temperate region (Germany) and the arid region (Egypt). *Environmental Pollution*, *262*. 10.1016/j.envpol.2020.114312 10.1016/j.envpol.2020.114312 32193081

[CR41] Shi P, Xiao J, Wang Y, Chen L (2014). Assessment of ecological and human health risks of heavy metal contamination in agriculture soils disturbed by pipeline construction. International Journal of Environmental Research and Public Health.

[CR42] Simion, A. F., Lazar, M., & Drebenstedt, C. (2020). Behaviour of pollutants in the upper reaches of East Jiu River case study. *Mining Science and Technology**, **4*(3), 181–187. 10.17073/2500-0632-2019-3-181-187

[CR43] Soltani N, Keshavarzi B, Moore F, Sorooshian A, Ahmadi MR (2017). Distribution of potentially toxic elements (PTEs) in tailings, soils, and plants around Gol-E-Gohar iron mine, a case study in Iran. Environmental Science and Pollution Research.

[CR44] Tóth G, Hermann T, Da Silva MR, Montanarella L (2016). Heavy metals in agricultural soils of the European Union with implications for food safety. Environment International.

[CR45] Tóth G, Hermann T, Szatmári G, Pásztor L (2016). Maps of heavy metals in the soils of the European Union and proposed priority areas for detailed assessment. Science of the Total Environment.

[CR46] Xiao R, Wang S, Li R, Wang JJ, Zhang Z (2017). Soil heavy metal contamination and health risks associated with artisanal gold mining in Tongguan, Shaanxi. China. Ecotoxicology and Environmental Safety.

[CR47] Zhang Z, Wang Q, Zheng D, Zheng N, Lu X (2010). Mercury distribution and bioaccumulation up the soil-plant-grasshopper-spider food chain in Huludao City. China. Journal of Environmental Sciences (china).

