# Supplementary material for: Artisanal gold mine spoil types within a common geological area and their variations in contaminant loads and human health risks
Source: Environ Monit Assess. 2023 Jan 20;195(2):312. doi: 10.1007/s10661-023-10932-4 (PMC9852104; doi:10.1007/s10661-023-10932-4)
Supplement: Supplementary file 1 — Supplementary file1 (ZIP 1654 KB) [file 10661_2023_10932_MOESM1_ESM.zip › Supplementary data/Nonparametric analysis.docx]

**Nonparametric Tests**

| **Notes** | | |
| --- | --- | --- |
| Output Created | | 29-DEC-2022 15:14:21 |
| Comments | |  |
| Input | Data | /Users/test/Desktop/Manuscripts/2022 manuscripts/spss analysed data/PTE variation SPSS/SPSS data 1.sav |
|  | Active Dataset | DataSet1 |
|  | Filter | <none> |
|  | Weight | <none> |
|  | Split File | <none> |
|  | N of Rows in Working Data File | 111 |
| Syntax | | NPTESTS /INDEPENDENT TEST (Cadmium Zinc Lead Mercury Arsenic Iron Alumimium pH EC) GROUP (Source) /MISSING SCOPE=ANALYSIS USERMISSING=EXCLUDE /CRITERIA ALPHA=0.05 CILEVEL=95. |
| Resources | Processor Time | 00:00:04.32 |
|  | Elapsed Time | 00:00:04.00 |

[DataSet1] /Users/test/Desktop/Manuscripts/2022 manuscripts/spss analysed data/PTE variation SPSS/SPSS data 1.sav

| **Hypothesis Test Summary** | | | | |
| --- | --- | --- | --- | --- |
|  | Null Hypothesis | Test | Sig.^a,b^ | Decision |
| 1 | The distribution of Cadmium is the same across categories of Source. | Independent-Samples Kruskal-Wallis Test | <.001 | Reject the null hypothesis. |
| 2 | The distribution of Zinc is the same across categories of Source. | Independent-Samples Kruskal-Wallis Test | <.001 | Reject the null hypothesis. |
| 3 | The distribution of Lead is the same across categories of Source. | Independent-Samples Kruskal-Wallis Test | <.001 | Reject the null hypothesis. |
| 4 | The distribution of Mercury is the same across categories of Source. | Independent-Samples Kruskal-Wallis Test | <.001 | Reject the null hypothesis. |
| 5 | The distribution of Arsenic is the same across categories of Source. | Independent-Samples Kruskal-Wallis Test | <.001 | Reject the null hypothesis. |
| 6 | The distribution of Iron is the same across categories of Source. | Independent-Samples Kruskal-Wallis Test | <.001 | Reject the null hypothesis. |
| 7 | The distribution of Alumimium is the same across categories of Source. | Independent-Samples Kruskal-Wallis Test | <.001 | Reject the null hypothesis. |
| 8 | The distribution of pH is the same across categories of Source. | Independent-Samples Kruskal-Wallis Test | <.001 | Reject the null hypothesis. |
| 9 | The distribution of EC is the same across categories of Source. | Independent-Samples Kruskal-Wallis Test | <.001 | Reject the null hypothesis. |
| a. The significance level is .050. | | | | |
| b. Asymptotic significance is displayed. | | | | |

**Independent-Samples Kruskal-Wallis Test**

**Cadmium across Source**

| **Independent-Samples Kruskal-Wallis Test Summary** | |
| --- | --- |
| Total N | 111 |
| Test Statistic | 89.203^a^ |
| Degree Of Freedom | 3 |
| Asymptotic Sig.(2-sided test) | <.001 |
| a. The test statistic is adjusted for ties. | |

| **Pairwise Comparisons of Source** | | | | | |
| --- | --- | --- | --- | --- | --- |
| Sample 1-Sample 2 | Test Statistic | Std. Error | Std. Test Statistic | Sig. | Adj. Sig.^a^ |
| FS-AvS | -25.886 | 9.154 | -2.828 | .005 | .028 |
| FS-UrS | -66.802 | 9.154 | -7.297 | <.001 | .000 |
| FS-OxS | -73.636 | 9.154 | -8.044 | <.001 | .000 |
| AvS-UrS | 40.917 | 8.308 | 4.925 | <.001 | .000 |
| AvS-OxS | 47.750 | 8.308 | 5.748 | <.001 | .000 |
| UrS-OxS | -6.833 | 8.308 | -.823 | .411 | 1.000 |
| Each row tests the null hypothesis that the Sample 1 and Sample 2 distributions are the same. Asymptotic significances (2-sided tests) are displayed. The significance level is .050. | | | | | |
| a. Significance values have been adjusted by the Bonferroni correction for multiple tests. | | | | | |

**Zinc across Source**

| **Independent-Samples Kruskal-Wallis Test Summary** | |
| --- | --- |
| Total N | 111 |
| Test Statistic | 85.129^a^ |
| Degree Of Freedom | 3 |
| Asymptotic Sig.(2-sided test) | <.001 |
| a. The test statistic is adjusted for ties. | |

| **Pairwise Comparisons of Source** | | | | | |
| --- | --- | --- | --- | --- | --- |
| Sample 1-Sample 2 | Test Statistic | Std. Error | Std. Test Statistic | Sig. | Adj. Sig.^a^ |
| FS-AvS | -10.281 | 9.158 | -1.123 | .262 | 1.000 |
| FS-OxS | -55.814 | 9.158 | -6.095 | <.001 | .000 |
| FS-UrS | -67.281 | 9.158 | -7.347 | <.001 | .000 |
| AvS-OxS | 45.533 | 8.311 | 5.479 | <.001 | .000 |
| AvS-UrS | 57.000 | 8.311 | 6.859 | <.001 | .000 |
| OxS-UrS | 11.467 | 8.311 | 1.380 | .168 | 1.000 |
| Each row tests the null hypothesis that the Sample 1 and Sample 2 distributions are the same. Asymptotic significances (2-sided tests) are displayed. The significance level is .050. | | | | | |
| a. Significance values have been adjusted by the Bonferroni correction for multiple tests. | | | | | |

**Lead across Source**

| **Independent-Samples Kruskal-Wallis Test Summary** | |
| --- | --- |
| Total N | 111 |
| Test Statistic | 86.854^a^ |
| Degree Of Freedom | 3 |
| Asymptotic Sig.(2-sided test) | <.001 |
| a. The test statistic is adjusted for ties. | |

| **Pairwise Comparisons of Source** | | | | | |
| --- | --- | --- | --- | --- | --- |
| Sample 1-Sample 2 | Test Statistic | Std. Error | Std. Test Statistic | Sig. | Adj. Sig.^a^ |
| FS-AvS | -20.238 | 9.158 | -2.210 | .027 | .163 |
| FS-OxS | -67.388 | 9.158 | -7.359 | <.001 | .000 |
| FS-UrS | -67.421 | 9.158 | -7.362 | <.001 | .000 |
| AvS-OxS | 47.150 | 8.310 | 5.674 | <.001 | .000 |
| AvS-UrS | 47.183 | 8.310 | 5.678 | <.001 | .000 |
| OxS-UrS | .033 | 8.310 | .004 | .997 | 1.000 |
| Each row tests the null hypothesis that the Sample 1 and Sample 2 distributions are the same. Asymptotic significances (2-sided tests) are displayed. The significance level is .050. | | | | | |
| a. Significance values have been adjusted by the Bonferroni correction for multiple tests. | | | | | |

**Mercury across Source**

| **Independent-Samples Kruskal-Wallis Test Summary** | |
| --- | --- |
| Total N | 111 |
| Test Statistic | 61.368^a^ |
| Degree Of Freedom | 3 |
| Asymptotic Sig.(2-sided test) | <.001 |
| a. The test statistic is adjusted for ties. | |

| **Pairwise Comparisons of Source** | | | | | |
| --- | --- | --- | --- | --- | --- |
| Sample 1-Sample 2 | Test Statistic | Std. Error | Std. Test Statistic | Sig. | Adj. Sig.^a^ |
| FS-AvS | -36.664 | 9.157 | -4.004 | <.001 | .000 |
| FS-OxS | -49.164 | 9.157 | -5.369 | <.001 | .000 |
| FS-UrS | -70.364 | 9.157 | -7.684 | <.001 | .000 |
| AvS-OxS | 12.500 | 8.310 | 1.504 | .133 | .795 |
| AvS-UrS | 33.700 | 8.310 | 4.055 | <.001 | .000 |
| OxS-UrS | 21.200 | 8.310 | 2.551 | .011 | .064 |
| Each row tests the null hypothesis that the Sample 1 and Sample 2 distributions are the same. Asymptotic significances (2-sided tests) are displayed. The significance level is .050. | | | | | |
| a. Significance values have been adjusted by the Bonferroni correction for multiple tests. | | | | | |

**Arsenic across Source**

| **Independent-Samples Kruskal-Wallis Test Summary** | |
| --- | --- |
| Total N | 111 |
| Test Statistic | 84.443^a^ |
| Degree Of Freedom | 3 |
| Asymptotic Sig.(2-sided test) | <.001 |
| a. The test statistic is adjusted for ties. | |

| **Pairwise Comparisons of Source** | | | | | |
| --- | --- | --- | --- | --- | --- |
| Sample 1-Sample 2 | Test Statistic | Std. Error | Std. Test Statistic | Sig. | Adj. Sig.^a^ |
| FS-AvS | -13.681 | 9.158 | -1.494 | .135 | .811 |
| FS-OxS | -61.481 | 9.158 | -6.713 | <.001 | .000 |
| FS-UrS | -65.614 | 9.158 | -7.165 | <.001 | .000 |
| AvS-OxS | 47.800 | 8.311 | 5.752 | <.001 | .000 |
| AvS-UrS | 51.933 | 8.311 | 6.249 | <.001 | .000 |
| OxS-UrS | 4.133 | 8.311 | .497 | .619 | 1.000 |
| Each row tests the null hypothesis that the Sample 1 and Sample 2 distributions are the same. Asymptotic significances (2-sided tests) are displayed. The significance level is .050. | | | | | |
| a. Significance values have been adjusted by the Bonferroni correction for multiple tests. | | | | | |

**Iron across Source**

| **Independent-Samples Kruskal-Wallis Test Summary** | |
| --- | --- |
| Total N | 111 |
| Test Statistic | 87.906^a^ |
| Degree Of Freedom | 3 |
| Asymptotic Sig.(2-sided test) | <.001 |
| a. The test statistic is adjusted for ties. | |

| **Pairwise Comparisons of Source** | | | | | |
| --- | --- | --- | --- | --- | --- |
| Sample 1-Sample 2 | Test Statistic | Std. Error | Std. Test Statistic | Sig. | Adj. Sig.^a^ |
| FS-AvS | -25.843 | 9.158 | -2.822 | .005 | .029 |
| FS-UrS | -56.410 | 9.158 | -6.160 | <.001 | .000 |
| FS-OxS | -78.610 | 9.158 | -8.584 | <.001 | .000 |
| AvS-UrS | 30.567 | 8.311 | 3.678 | <.001 | .001 |
| AvS-OxS | 52.767 | 8.311 | 6.349 | <.001 | .000 |
| UrS-OxS | -22.200 | 8.311 | -2.671 | .008 | .045 |
| Each row tests the null hypothesis that the Sample 1 and Sample 2 distributions are the same. Asymptotic significances (2-sided tests) are displayed. The significance level is .050. | | | | | |
| a. Significance values have been adjusted by the Bonferroni correction for multiple tests. | | | | | |

**Alumimium across Source**

| **Independent-Samples Kruskal-Wallis Test Summary** | |
| --- | --- |
| Total N | 111 |
| Test Statistic | 84.652^a^ |
| Degree Of Freedom | 3 |
| Asymptotic Sig.(2-sided test) | <.001 |
| a. The test statistic is adjusted for ties. | |

| **Pairwise Comparisons of Source** | | | | | |
| --- | --- | --- | --- | --- | --- |
| Sample 1-Sample 2 | Test Statistic | Std. Error | Std. Test Statistic | Sig. | Adj. Sig.^a^ |
| FS-AvS | -24.138 | 9.158 | -2.636 | .008 | .050 |
| FS-UrS | -55.805 | 9.158 | -6.094 | <.001 | .000 |
| FS-OxS | -76.338 | 9.158 | -8.336 | <.001 | .000 |
| AvS-UrS | 31.667 | 8.311 | 3.810 | <.001 | .001 |
| AvS-OxS | 52.200 | 8.311 | 6.281 | <.001 | .000 |
| UrS-OxS | -20.533 | 8.311 | -2.471 | .013 | .081 |
| Each row tests the null hypothesis that the Sample 1 and Sample 2 distributions are the same. Asymptotic significances (2-sided tests) are displayed. The significance level is .050. | | | | | |
| a. Significance values have been adjusted by the Bonferroni correction for multiple tests. | | | | | |

**pH across Source**

| **Independent-Samples Kruskal-Wallis Test Summary** | |
| --- | --- |
| Total N | 111 |
| Test Statistic | 40.936^a^ |
| Degree Of Freedom | 3 |
| Asymptotic Sig.(2-sided test) | <.001 |
| a. The test statistic is adjusted for ties. | |

| **Pairwise Comparisons of Source** | | | | | |
| --- | --- | --- | --- | --- | --- |
| Sample 1-Sample 2 | Test Statistic | Std. Error | Std. Test Statistic | Sig. | Adj. Sig.^a^ |
| UrS-OxS | -23.133 | 8.310 | -2.784 | .005 | .032 |
| UrS-AvS | -32.900 | 8.310 | -3.959 | <.001 | .000 |
| UrS-FS | 57.205 | 9.157 | 6.247 | <.001 | .000 |
| OxS-AvS | -9.767 | 8.310 | -1.175 | .240 | 1.000 |
| OxS-FS | 34.071 | 9.157 | 3.721 | <.001 | .001 |
| AvS-FS | 24.305 | 9.157 | 2.654 | .008 | .048 |
| Each row tests the null hypothesis that the Sample 1 and Sample 2 distributions are the same. Asymptotic significances (2-sided tests) are displayed. The significance level is .050. | | | | | |
| a. Significance values have been adjusted by the Bonferroni correction for multiple tests. | | | | | |

**EC across Source**

| **Independent-Samples Kruskal-Wallis Test Summary** | |
| --- | --- |
| Total N | 111 |
| Test Statistic | 88.328^a^ |
| Degree Of Freedom | 3 |
| Asymptotic Sig.(2-sided test) | <.001 |
| a. The test statistic is adjusted for ties. | |

| **Pairwise Comparisons of Source** | | | | | |
| --- | --- | --- | --- | --- | --- |
| Sample 1-Sample 2 | Test Statistic | Std. Error | Std. Test Statistic | Sig. | Adj. Sig.^a^ |
| FS-AvS | -22.060 | 9.153 | -2.410 | .016 | .096 |
| FS-UrS | -65.660 | 9.153 | -7.174 | <.001 | .000 |
| FS-OxS | -71.293 | 9.153 | -7.789 | <.001 | .000 |
| AvS-UrS | 43.600 | 8.306 | 5.249 | <.001 | .000 |
| AvS-OxS | 49.233 | 8.306 | 5.928 | <.001 | .000 |
| UrS-OxS | -5.633 | 8.306 | -.678 | .498 | 1.000 |
| Each row tests the null hypothesis that the Sample 1 and Sample 2 distributions are the same. Asymptotic significances (2-sided tests) are displayed. The significance level is .050. | | | | | |
| a. Significance values have been adjusted by the Bonferroni correction for multiple tests. | | | | | |

**Nonparametric Tests**

| **Notes** | | |
| --- | --- | --- |
| Output Created | | 29-DEC-2022 17:26:00 |
| Comments | |  |
| Input | Data | /Users/test/Desktop/Manuscripts/2022 manuscripts/spss analysed data/PTE variation SPSS/Non parametric test.sav |
|  | Active Dataset | DataSet1 |
|  | Filter | <none> |
|  | Weight | <none> |
|  | Split File | <none> |
|  | N of Rows in Working Data File | 111 |
| Syntax | | NPTESTS /INDEPENDENT TEST (Cadmium Zinc Lead Mercury Arsenic Iron Alumimium pH EC) GROUP (Source) /MISSING SCOPE=ANALYSIS USERMISSING=EXCLUDE /CRITERIA ALPHA=0.05 CILEVEL=95. |
| Resources | Processor Time | 00:00:04.30 |
|  | Elapsed Time | 00:00:04.00 |

[DataSet1] /Users/test/Desktop/Manuscripts/2022 manuscripts/spss analysed data/PTE variation SPSS/Non parametric test.sav

| **Hypothesis Test Summary** | | | | |
| --- | --- | --- | --- | --- |
|  | Null Hypothesis | Test | Sig.^a,b^ | Decision |
| 1 | The distribution of Cadmium is the same across categories of Source. | Independent-Samples Kruskal-Wallis Test | <.001 | Reject the null hypothesis. |
| 2 | The distribution of Zinc is the same across categories of Source. | Independent-Samples Kruskal-Wallis Test | <.001 | Reject the null hypothesis. |
| 3 | The distribution of Lead is the same across categories of Source. | Independent-Samples Kruskal-Wallis Test | <.001 | Reject the null hypothesis. |
| 4 | The distribution of Mercury is the same across categories of Source. | Independent-Samples Kruskal-Wallis Test | <.001 | Reject the null hypothesis. |
| 5 | The distribution of Arsenic is the same across categories of Source. | Independent-Samples Kruskal-Wallis Test | <.001 | Reject the null hypothesis. |
| 6 | The distribution of Iron is the same across categories of Source. | Independent-Samples Kruskal-Wallis Test | <.001 | Reject the null hypothesis. |
| 7 | The distribution of Alumimium is the same across categories of Source. | Independent-Samples Kruskal-Wallis Test | <.001 | Reject the null hypothesis. |
| 8 | The distribution of pH is the same across categories of Source. | Independent-Samples Kruskal-Wallis Test | <.001 | Reject the null hypothesis. |
| 9 | The distribution of EC is the same across categories of Source. | Independent-Samples Kruskal-Wallis Test | <.001 | Reject the null hypothesis. |
| a. The significance level is .050. | | | | |
| b. Asymptotic significance is displayed. | | | | |

**Independent-Samples Kruskal-Wallis Test**

**Cadmium across Source**

| **Independent-Samples Kruskal-Wallis Test Summary** | |
| --- | --- |
| Total N | 111 |
| Test Statistic | 89.203^a^ |
| Degree Of Freedom | 3 |
| Asymptotic Sig.(2-sided test) | <.001 |
| a. The test statistic is adjusted for ties. | |

| **Pairwise Comparisons of Source** | | | | | |
| --- | --- | --- | --- | --- | --- |
| Sample 1-Sample 2 | Test Statistic | Std. Error | Std. Test Statistic | Sig. | Adj. Sig.^a^ |
| FS-AVS | -25.886 | 9.154 | -2.828 | .005 | .028 |
| FS-URS | -66.802 | 9.154 | -7.297 | <.001 | .000 |
| FS-OXS | -73.636 | 9.154 | -8.044 | <.001 | .000 |
| AVS-URS | 40.917 | 8.308 | 4.925 | <.001 | .000 |
| AVS-OXS | 47.750 | 8.308 | 5.748 | <.001 | .000 |
| URS-OXS | -6.833 | 8.308 | -.823 | .411 | 1.000 |
| Each row tests the null hypothesis that the Sample 1 and Sample 2 distributions are the same. Asymptotic significances (2-sided tests) are displayed. The significance level is .050. | | | | | |
| a. Significance values have been adjusted by the Bonferroni correction for multiple tests. | | | | | |

**Zinc across Source**

| **Independent-Samples Kruskal-Wallis Test Summary** | |
| --- | --- |
| Total N | 111 |
| Test Statistic | 85.129^a^ |
| Degree Of Freedom | 3 |
| Asymptotic Sig.(2-sided test) | <.001 |
| a. The test statistic is adjusted for ties. | |

| **Pairwise Comparisons of Source** | | | | | |
| --- | --- | --- | --- | --- | --- |
| Sample 1-Sample 2 | Test Statistic | Std. Error | Std. Test Statistic | Sig. | Adj. Sig.^a^ |
| FS-AVS | -10.281 | 9.158 | -1.123 | .262 | 1.000 |
| FS-OXS | -55.814 | 9.158 | -6.095 | <.001 | .000 |
| FS-URS | -67.281 | 9.158 | -7.347 | <.001 | .000 |
| AVS-OXS | 45.533 | 8.311 | 5.479 | <.001 | .000 |
| AVS-URS | 57.000 | 8.311 | 6.859 | <.001 | .000 |
| OXS-URS | 11.467 | 8.311 | 1.380 | .168 | 1.000 |
| Each row tests the null hypothesis that the Sample 1 and Sample 2 distributions are the same. Asymptotic significances (2-sided tests) are displayed. The significance level is .050. | | | | | |
| a. Significance values have been adjusted by the Bonferroni correction for multiple tests. | | | | | |

**Lead across Source**

| **Independent-Samples Kruskal-Wallis Test Summary** | |
| --- | --- |
| Total N | 111 |
| Test Statistic | 86.854^a^ |
| Degree Of Freedom | 3 |
| Asymptotic Sig.(2-sided test) | <.001 |
| a. The test statistic is adjusted for ties. | |

| **Pairwise Comparisons of Source** | | | | | |
| --- | --- | --- | --- | --- | --- |
| Sample 1-Sample 2 | Test Statistic | Std. Error | Std. Test Statistic | Sig. | Adj. Sig.^a^ |
| FS-AVS | -20.238 | 9.158 | -2.210 | .027 | .163 |
| FS-OXS | -67.388 | 9.158 | -7.359 | <.001 | .000 |
| FS-URS | -67.421 | 9.158 | -7.362 | <.001 | .000 |
| AVS-OXS | 47.150 | 8.310 | 5.674 | <.001 | .000 |
| AVS-URS | 47.183 | 8.310 | 5.678 | <.001 | .000 |
| OXS-URS | .033 | 8.310 | .004 | .997 | 1.000 |
| Each row tests the null hypothesis that the Sample 1 and Sample 2 distributions are the same. Asymptotic significances (2-sided tests) are displayed. The significance level is .050. | | | | | |
| a. Significance values have been adjusted by the Bonferroni correction for multiple tests. | | | | | |

**Mercury across Source**

| **Independent-Samples Kruskal-Wallis Test Summary** | |
| --- | --- |
| Total N | 111 |
| Test Statistic | 61.368^a^ |
| Degree Of Freedom | 3 |
| Asymptotic Sig.(2-sided test) | <.001 |
| a. The test statistic is adjusted for ties. | |

| **Pairwise Comparisons of Source** | | | | | |
| --- | --- | --- | --- | --- | --- |
| Sample 1-Sample 2 | Test Statistic | Std. Error | Std. Test Statistic | Sig. | Adj. Sig.^a^ |
| FS-AVS | -36.664 | 9.157 | -4.004 | <.001 | .000 |
| FS-OXS | -49.164 | 9.157 | -5.369 | <.001 | .000 |
| FS-URS | -70.364 | 9.157 | -7.684 | <.001 | .000 |
| AVS-OXS | 12.500 | 8.310 | 1.504 | .133 | .795 |
| AVS-URS | 33.700 | 8.310 | 4.055 | <.001 | .000 |
| OXS-URS | 21.200 | 8.310 | 2.551 | .011 | .064 |
| Each row tests the null hypothesis that the Sample 1 and Sample 2 distributions are the same. Asymptotic significances (2-sided tests) are displayed. The significance level is .050. | | | | | |
| a. Significance values have been adjusted by the Bonferroni correction for multiple tests. | | | | | |

**Arsenic across Source**

| **Independent-Samples Kruskal-Wallis Test Summary** | |
| --- | --- |
| Total N | 111 |
| Test Statistic | 84.443^a^ |
| Degree Of Freedom | 3 |
| Asymptotic Sig.(2-sided test) | <.001 |
| a. The test statistic is adjusted for ties. | |

| **Pairwise Comparisons of Source** | | | | | |
| --- | --- | --- | --- | --- | --- |
| Sample 1-Sample 2 | Test Statistic | Std. Error | Std. Test Statistic | Sig. | Adj. Sig.^a^ |
| FS-AVS | -13.681 | 9.158 | -1.494 | .135 | .811 |
| FS-OXS | -61.481 | 9.158 | -6.713 | <.001 | .000 |
| FS-URS | -65.614 | 9.158 | -7.165 | <.001 | .000 |
| AVS-OXS | 47.800 | 8.311 | 5.752 | <.001 | .000 |
| AVS-URS | 51.933 | 8.311 | 6.249 | <.001 | .000 |
| OXS-URS | 4.133 | 8.311 | .497 | .619 | 1.000 |
| Each row tests the null hypothesis that the Sample 1 and Sample 2 distributions are the same. Asymptotic significances (2-sided tests) are displayed. The significance level is .050. | | | | | |
| a. Significance values have been adjusted by the Bonferroni correction for multiple tests. | | | | | |

**Iron across Source**

| **Independent-Samples Kruskal-Wallis Test Summary** | |
| --- | --- |
| Total N | 111 |
| Test Statistic | 87.906^a^ |
| Degree Of Freedom | 3 |
| Asymptotic Sig.(2-sided test) | <.001 |
| a. The test statistic is adjusted for ties. | |

| **Pairwise Comparisons of Source** | | | | | |
| --- | --- | --- | --- | --- | --- |
| Sample 1-Sample 2 | Test Statistic | Std. Error | Std. Test Statistic | Sig. | Adj. Sig.^a^ |
| FS-AVS | -25.843 | 9.158 | -2.822 | .005 | .029 |
| FS-URS | -56.410 | 9.158 | -6.160 | <.001 | .000 |
| FS-OXS | -78.610 | 9.158 | -8.584 | <.001 | .000 |
| AVS-URS | 30.567 | 8.311 | 3.678 | <.001 | .001 |
| AVS-OXS | 52.767 | 8.311 | 6.349 | <.001 | .000 |
| URS-OXS | -22.200 | 8.311 | -2.671 | .008 | .045 |
| Each row tests the null hypothesis that the Sample 1 and Sample 2 distributions are the same. Asymptotic significances (2-sided tests) are displayed. The significance level is .050. | | | | | |
| a. Significance values have been adjusted by the Bonferroni correction for multiple tests. | | | | | |

**Alumimium across Source**

| **Independent-Samples Kruskal-Wallis Test Summary** | |
| --- | --- |
| Total N | 111 |
| Test Statistic | 84.652^a^ |
| Degree Of Freedom | 3 |
| Asymptotic Sig.(2-sided test) | <.001 |
| a. The test statistic is adjusted for ties. | |

| **Pairwise Comparisons of Source** | | | | | |
| --- | --- | --- | --- | --- | --- |
| Sample 1-Sample 2 | Test Statistic | Std. Error | Std. Test Statistic | Sig. | Adj. Sig.^a^ |
| FS-AVS | -24.138 | 9.158 | -2.636 | .008 | .050 |
| FS-URS | -55.805 | 9.158 | -6.094 | <.001 | .000 |
| FS-OXS | -76.338 | 9.158 | -8.336 | <.001 | .000 |
| AVS-URS | 31.667 | 8.311 | 3.810 | <.001 | .001 |
| AVS-OXS | 52.200 | 8.311 | 6.281 | <.001 | .000 |
| URS-OXS | -20.533 | 8.311 | -2.471 | .013 | .081 |
| Each row tests the null hypothesis that the Sample 1 and Sample 2 distributions are the same. Asymptotic significances (2-sided tests) are displayed. The significance level is .050. | | | | | |
| a. Significance values have been adjusted by the Bonferroni correction for multiple tests. | | | | | |

**pH across Source**

| **Independent-Samples Kruskal-Wallis Test Summary** | |
| --- | --- |
| Total N | 111 |
| Test Statistic | 40.936^a^ |
| Degree Of Freedom | 3 |
| Asymptotic Sig.(2-sided test) | <.001 |
| a. The test statistic is adjusted for ties. | |

| **Pairwise Comparisons of Source** | | | | | |
| --- | --- | --- | --- | --- | --- |
| Sample 1-Sample 2 | Test Statistic | Std. Error | Std. Test Statistic | Sig. | Adj. Sig.^a^ |
| URS-OXS | -23.133 | 8.310 | -2.784 | .005 | .032 |
| URS-AVS | -32.900 | 8.310 | -3.959 | <.001 | .000 |
| URS-FS | 57.205 | 9.157 | 6.247 | <.001 | .000 |
| OXS-AVS | -9.767 | 8.310 | -1.175 | .240 | 1.000 |
| OXS-FS | 34.071 | 9.157 | 3.721 | <.001 | .001 |
| AVS-FS | 24.305 | 9.157 | 2.654 | .008 | .048 |
| Each row tests the null hypothesis that the Sample 1 and Sample 2 distributions are the same. Asymptotic significances (2-sided tests) are displayed. The significance level is .050. | | | | | |
| a. Significance values have been adjusted by the Bonferroni correction for multiple tests. | | | | | |

**EC across Source**

| **Independent-Samples Kruskal-Wallis Test Summary** | |
| --- | --- |
| Total N | 111 |
| Test Statistic | 88.328^a^ |
| Degree Of Freedom | 3 |
| Asymptotic Sig.(2-sided test) | <.001 |
| a. The test statistic is adjusted for ties. | |

| **Pairwise Comparisons of Source** | | | | | |
| --- | --- | --- | --- | --- | --- |
| Sample 1-Sample 2 | Test Statistic | Std. Error | Std. Test Statistic | Sig. | Adj. Sig.^a^ |
| FS-AVS | -22.060 | 9.153 | -2.410 | .016 | .096 |
| FS-URS | -65.660 | 9.153 | -7.174 | <.001 | .000 |
| FS-OXS | -71.293 | 9.153 | -7.789 | <.001 | .000 |
| AVS-URS | 43.600 | 8.306 | 5.249 | <.001 | .000 |
| AVS-OXS | 49.233 | 8.306 | 5.928 | <.001 | .000 |
| URS-OXS | -5.633 | 8.306 | -.678 | .498 | 1.000 |
| Each row tests the null hypothesis that the Sample 1 and Sample 2 distributions are the same. Asymptotic significances (2-sided tests) are displayed. The significance level is .050. | | | | | |
| a. Significance values have been adjusted by the Bonferroni correction for multiple tests. | | | | | |
