# Supplementary material for: Artisanal gold mine spoil types within a common geological area and their variations in contaminant loads and human health risks
Source: Environ Monit Assess. 2023 Jan 20;195(2):312. doi: 10.1007/s10661-023-10932-4 (PMC9852104; doi:10.1007/s10661-023-10932-4)
Supplement: Supplementary file 1 — Supplementary file1 (ZIP 1654 KB) [file 10661_2023_10932_MOESM1_ESM.zip › Supplementary data/Supplementary Tables.docx]

Supplementary Tables

Table A.1: Mean and Standard deviation values of the chemical properties of the studied areas.

| **Sample source** | **Sample**  **Size (n)** | **Mean ± Standard Deviation** | | | | | | | | |
| --- | --- | --- | --- | --- | --- | --- | --- | --- | --- | --- |
|  |  | **Cadmium** | **Zinc** | **Lead** | **Mercury** | **Arsenic** | **Iron** | **Aluminium** | **pH** | **Electronic conductivity** |
| Forest | 20 | 0.1 **±** 0.2 | 19.6 **±** 5.9 | 2.6 **±** 2.0 | 0.1 **±** 0.2 | 5.9 **±** 4.3 | 5974.1 **±** 1529.1 | 2644.0 **±** 659.6 | 5.9 **±** 0.8 | 48.5 **±** 24.5 |
| Underground rock spoil (UrS) | 30 | 9.5 **±** 4.8 | 226.1 **±** 78.6 | 17.8 **±** 4.7 | 3.8 **±** 2.0 | 282.8 **±** 72.9 | 14048.1 **±** 5223.6 | 6404.8 **±** 2859.0 | 4.3 **±** 0.6 | 1696.4 **±** 241.0 |
| Oxide spoils (OxS) | 30 | 10.0 **±** 3.0 | 175.7 **±** 102.8 | 17.7 **±** 6.9 | 2.2 **±** 1.9 | 260.2 **±** 115.4 | 45100.4 **±** 9567.0 | 20589.5 **±** 5616.1 | 4.9 **±** 0.7 | 1786.0 **±** 142.1 |
| Alluvial spoils (AvS) | 30 | 2.8 **±** 1.6 | 23.0 **±** 2.2 | 5.2 **±** 1.0 | 1.4 **±** 1.3 | 9.6 **±** 2.1 | 31360.6 **±** 11339.6 | 14407.3 **±** 5668.5 | 5.1 **±** 0.7 | 104.0 **±** 31.9 |

Table A.2: Potential Average Daily Dose (ADD) estimation for children.

| Sample source | Potentially Toxic Elements (mg/kg) | | | | |
| --- | --- | --- | --- | --- | --- |
|  | Cd | As | Pb | Hg | Zn |
| FS | 0.00 | 0.00 | 0.00 | 0.00 | 0.00 |
| UrS | 0.00 | 0.02 | 0.00 | 0.00 | 0.02 |
| OxS | 0.00 | 0.02 | 0.00 | 0.00 | 0.01 |
| AvS | 0.00 | 0.00 | 0.00 | 0.00 | 0.00 |

Table A.3: Potential Average Daily Dose (ADD) estimation for matured females.

| Sample source | Potentially Toxic Elements (mg/kg) | | | | |
| --- | --- | --- | --- | --- | --- |
|  | Cd | As | Pb | Hg | Zn |
| FS | 7.1E-07 | 4.2E-05 | 1.8E-05 | 9.9E-07 | 1.4E-04 |
| UrS | 6.7E-05 | 2.0E-03 | 1.3E-04 | 2.7E-05 | 1.6E-03 |
| OxS | 7.1E-05 | 1.8E-03 | 1.3E-04 | 1.6E-05 | 1.2E-03 |
| AvS | 2.0E-05 | 6.8E-05 | 3.7E-05 | 9.6E-06 | 1.6E-04 |

Table A.4: Potential Average Daily Dose (ADD) estimation for matured males

| Sample source | Potentially Toxic Elements (mg/kg) | | | | |
| --- | --- | --- | --- | --- | --- |
|  | Cd | As | Pb | Hg | Zn |
| FS | 6.0E-07 | 3.6E-05 | 1.6E-05 | 8.5E-07 | 1.2E-04 |
| UrS | 5.7E-05 | 1.7E-03 | 1.1E-04 | 2.3E-05 | 1.4E-03 |
| OxS | 6.1E-05 | 1.6E-03 | 1.1E-04 | 1.3E-05 | 1.1E-03 |
| AvS | 1.7E-05 | 5.8E-05 | 3.1E-05 | 8.2E-06 | 1.4E-04 |
